# Endoscopic Endonasal Resection of Meckel's Cave Epidermoid Cysts: Case Discussion and Literature Review

**DOI:** 10.1155/2020/7853279

**Published:** 2020-02-07

**Authors:** Jehad Zakaria, Pravesh Saini, Mariya Yanovskaya, John T. Tsiang, Krishnan Ravindran, Stephen Johans, Chirag R. Patel, Anand V. Germanwala

**Affiliations:** ^1^Department of Neurological Surgery, Stritch School of Medicine, Loyola University Medical Center, Maywood, IL, USA; ^2^Department of Otolaryngology-Head and Neck Surgery, Stritch School of Medicine, Loyola University Medical Center, Maywood, IL, USA

## Abstract

Meckel's cave (MC) epidermoid cysts are relatively uncommon lesions. In cases where surgical excision is indicated, resection is often carried out via a frontosphenotemporal craniotomy from an anterolateral approach or a temporal craniotomy with or without a petrosectomy for a lateral corridor; both of these routes are associated with brain retraction and potential neurovascular injury. The anterior location of MC in the middle cranial fossa makes safe access via posterior fossa-based approaches—such as the retrosigmoid approach—challenging as well. Here, we present the cases of two patients diagnosed with epidermoid cysts in MC who underwent surgical resection via an endoscopic endonasal transpterygoid approach. Near-total resection was achieved in both cases, with only mild transient neurologic disturbances postoperatively. Radiographically, no evidence of residual disease was noted in either patient. We further review the nuances of an extended endoscopic endonasal approach to these lesions.

## 1. Introduction

Epidermoids are cystic lesions that are generally considered to be benign lesions that occupy the CSF space. On magnetic resonance imaging (MRI), they characteristically appear hypointense on T1-weighted and hyperintense on T2-weighted sequences [1, 2]. Notably, these lesions restrict diffusion of water and thus appear hyperintense on diffuse-weighted imaging. Advocated treatment of epidermoids of MC depends on the extent of symptom profile [3]. Open surgical approaches, including extradural exposure via a pterional or temporal craniotomy (with or without a petrosectomy), require significant retraction and place at risk a number of critical neurovascular structures [4]. Posterior fossa approaches, such as the retrosigmoid corridor, are challenging given the anterior extent of MC within the middle fossa and the resultant long working corridor. Novel extension of the endoscopic endonasal approach to the middle cranial fossa carries significant promise to reach these and other lesions within MC with minimal brain retraction [5, 6]. Here, we describe the successful use of the endoscopic endonasal approach to treat two patients with epidermoid cysts in MC.

## 2. Case Presentations

### 2.1. Case 1

**Figure 1:**
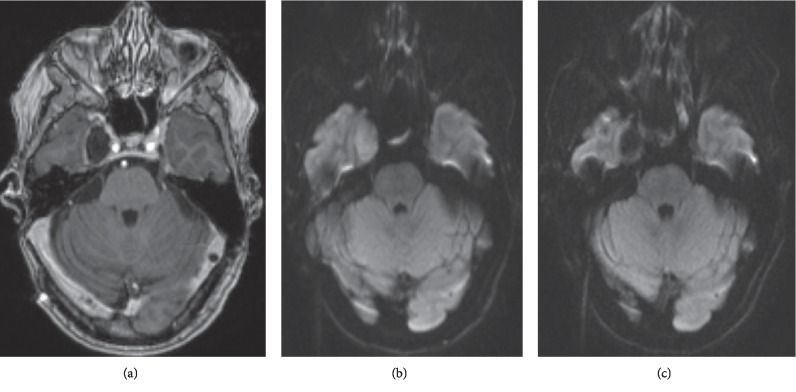
Imaging of patient 1. (a) Preoperative contrast-enhanced axial T1 MRI showing peripheral enhancement of the cyst wall. (b) Diffusion restriction noted in the lesion preoperatively. (c) Postoperative resolution of diffusion restriction within the lesion.

A 64-year-old woman was sent to our clinic for evaluation of a 2-year history of right-sided facial numbness. Her symptoms involved all divisions of the trigeminal nerve. She had initially presented to our institution 12 years prior with an MRI of the brain revealing both a large right cerebellopontine angle mass, suggestive of a vestibular schwannoma, as well as a right middle cranial fossa lesion radiographically consistent with an epidermoid cyst. She underwent resection of the vestibular schwannoma at our institution and remained asymptomatic during several years of clinical follow-up. Ten years following resection of the vestibular schwannoma, she began to experience progressive right-sided facial numbness, which persisted for two years. MRI of the brain at this time revealed mild radiographic progression of her known right-sided MC lesion, measuring 2.1 × 1.3 cm in greatest dimensions (Figure 1). Given the lesion growth and the progressive neurologic deficit, surgical intervention was considered and discussed with the patient. She underwent an endoscopic endonasal right transpterygoid approach to the cyst. CN VI was thought to be displaced medially and superiorly by the lesion and was not encountered. Successful evacuation of the cyst contents was achieved with histopathologic confirmation of an epidermoid. A CSF leak was encountered and closed in layers with a collagen-based duraplasty graft followed by a free mucosal graft. The patient's postoperative course was notable for intermittent pain in the right V2 distribution that gradually resolved over a period of several weeks. She was seen in the clinic after six weeks with continued improvement in her symptoms and remains stable from a clinical and imaging standpoint after one year.

### 2.2. Case 2

**Figure 2:**
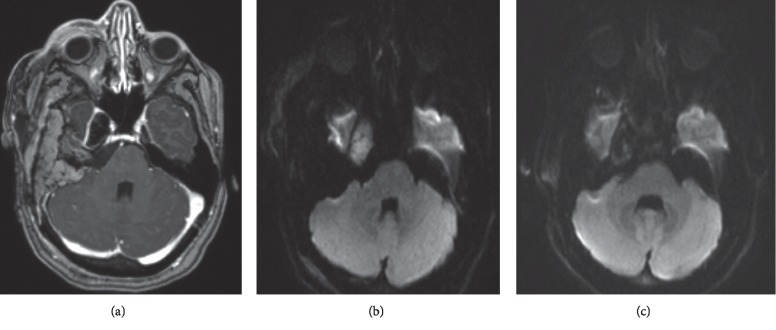
Imaging of patient 2. (a) Preoperative contrast-enhanced axial T1 MRI, showing peripheral enhancement of the cyst wall. (b) Diffusion restriction noted in the lesion preoperatively. (c) Postoperative resolution of diffusion restriction within the lesion.

**Figure 3:**
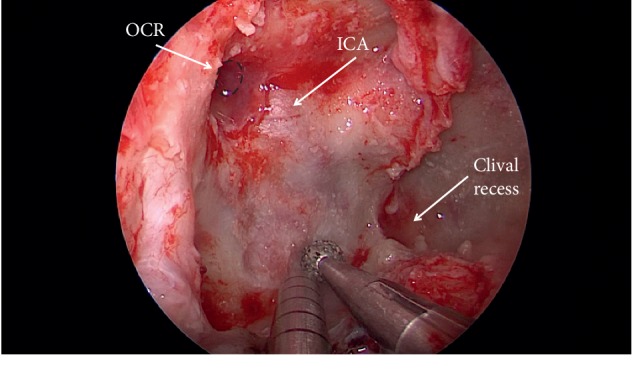
Intraoperative photo demonstrating drilling of the skull base through the endoscopic endonasal transpterygoid corridor.

**Figure 4:**
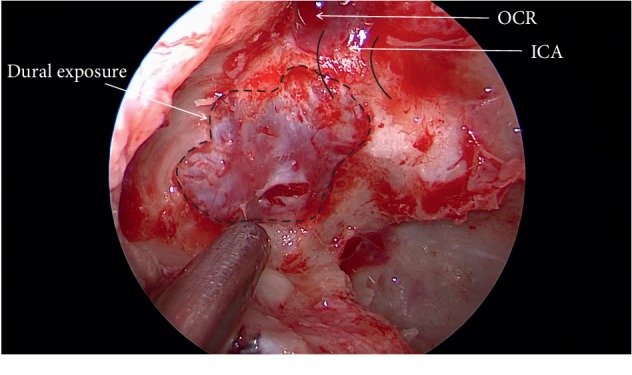
Exposure and opening of the dura.

**Figure 5:**
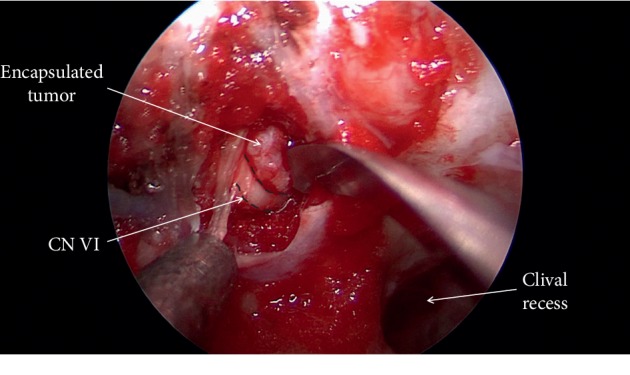
Visualization of the encapsulated epidermoid in a corridor superomedial to the abducens nerve.

**Figure 6:**
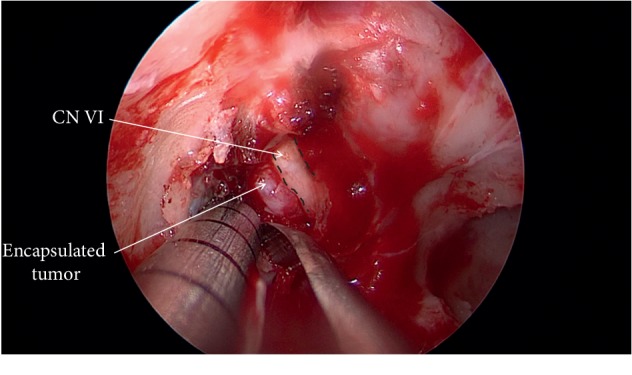
Visualization of the encapsulated epidermoid in a corridor inferolateral to the abducens nerve.

**Figure 7:**
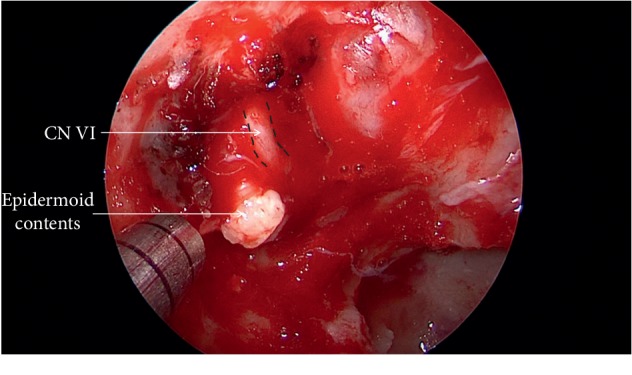
Visualization of the typical epidermoid contents following capsular opening inferolateral to the abducens nerve.

**Figure 8:**
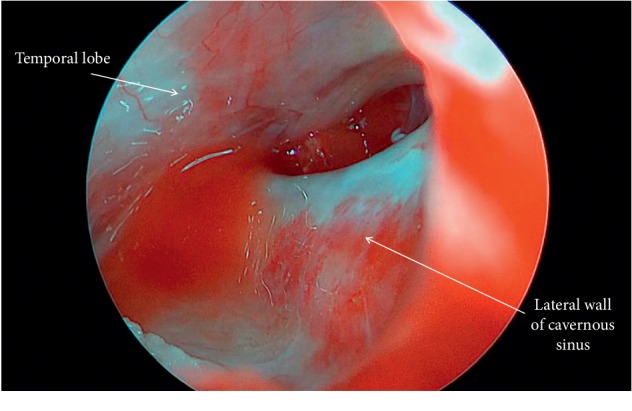
Intraoperative photo of Meckel's cave following epidermoid resection. The endoscope was advanced into the dural opening for visualization of Meckel's cave. The medial wall of Meckel's cave is contiguous with the lateral wall of the cavernous sinus. Temporal lobe is noted superolaterally.

A 26-year-old woman with a history of congenital bilateral sensorineural hearing loss was found to have a large right-sided cerebellopontine angle mass extending anteriorly to the middle cranial fossa into the region of MC. The patient had previously undergone a right temporal and petrosal craniotomy for resection of this mass; histopathological analysis at that time demonstrated an epidermoid lesion. Given technical difficulties in resecting the anterior most aspect of the epidermoid via an open craniotomy, an endoscopic endonasal right transpterygoid approach to the remaining epidermoid cyst in MC was undertaken (Figures 2–4). CN VI was visualized directly over the cyst capsule requiring evacuation of cyst contents through windows both superomedial and inferolateral to it (Figures 5–7). Intermittent EMG activity was noted during the procedure, and the nerve stimulated normally at the end of the surgery. Visualization of the resection cavity within MC following the resection revealed the temporal lobe superolaterally and the lateral wall of the cavernous sinus medially (Figure 8). The defect was reconstructed in layers with a collagen-based duraplasty graft followed by a nasoseptal flap from the contralateral nasal cavity. Her postoperative course was notable for a transient partial abducens palsy that had resolved upon six-week follow-up. She remains stable from a clinical and imaging standpoint after one year.

### 2.3. Technical Note

The endoscopic endonasal transpterygoid approach has been well described previously and is beyond the scope of this paper [7]. Both patients underwent a standard right transpterygoid approach with neuromonitoring of somatosensory evoked potentials (SSEPs), electroencephalogram (EEG), and electromyography (EMG) of the masseter and lateral rectus muscle for trigeminal and abducens nerve monitoring, respectively.

## 3. Discussion

MC is a dural recess in the posteromedial portion of the middle cranial fossa. Lesions in this region are uncommon overall and can be varied in their etiologies [8]. Space-occupying lesions in this CSF-containing subarachnoid space can present with a variety of signs and symptoms, related to neurovascular structures within the cave and adjacent to it. Namely, compression of the gasserian ganglion or any of the trigeminal nerve branches in this region can readily lead to trigeminal distribution pain, numbness, or diminished sensation [9–11]. Additionally, extension of the compressive lesions beyond MC can cause compression of other cranial nerves, causing, among others, facial nerve palsy and oculomotor nerve palsy.

The PubMed electronic database was searched from inception until December 2019 for English language studies using the search terms “Meckel's” and “epidermoid.” A total of 25 peer-reviewed articles were identified, reviewed, and included in our analysis. No articles were excluded. Only one of these 25 articles describes one patient with an epidermoid in a series of 8 patients with Meckel's cave tumors undergoing the endoscopic endonasal approach, substantiating the limited literature available on this topic [12]. Epidermoid cysts in MC are exceedingly rare and to our knowledge have only been described by a handful of case reports, with symptom profiles ranging from completely asymptomatic to severe compression causing trigeminal neuralgia [8, 13–15]. Indeed, in one of the largest series to date evaluating outcomes following surgical excision of MC tumors, out of twelve patients, only one epidermoid cyst was diagnosed [16]. The most common intrinsic lesions known to occur in MC are trigeminal schwannomas and meningiomas, among others [2, 6, 16]. Intracranial epidermoid cysts, on the other hand, are generally located extra-axially, with a predilection for the cerebellopontine angle and suprasellar regions, though intraparenchymal epidermoid cysts have also been described [13, 16].

The most commonly used surgical approach to MC has been the pterional approach, providing anterolateral access to MC but concomitantly associated with risk of damage to traversing neurovascular structures along a narrow operative corridor bounded by the internal carotid artery, the petrous apex, geniculate ganglion, and trigeminal nerve branches, amongst others [17, 18]. Furthermore, adequate exposure of MC may be limited with certain subtemporal/petrosal approaches, notwithstanding the need for temporal lobe retraction [19]. Other frequently used surgical approaches include the retrosigmoid approach. Safain et al. reported use of an endoscopic-assisted retrosigmoid approach to an epidermoid cyst at the cerebellopontine angle with extension into MC [20]. Although this technique is commonly used on tumors found at the cerebellopontine angle and allows proper exposure to the space, it should be noted that long working corridor to MC through the retrosigmoid approach adds to the risk of neurovascular injury [21].

The endoscopic endonasal approach to the cranial base has undergone a tremendous transformation over the past few decades, heralded by the advent of newer more sophisticated tools and instruments to allow access to the region. Careful consideration of anatomic corridors along with appropriate patient selection continues to allow surgeons to expand the use of the endoscope in addressing more complex regions in the anterior cranial fossa [22, 23]. The lateral location of MC provides technical challenges to the endoscopic approach, given the difficult working trajectory as opposed to the straight endoscopic trajectory of approaches to the sellar region. Additional working corridors, such as the transmaxillary corridor via Caldwell-Luc, can be considered for access to more lateral structures [24].

**Figure 9:**
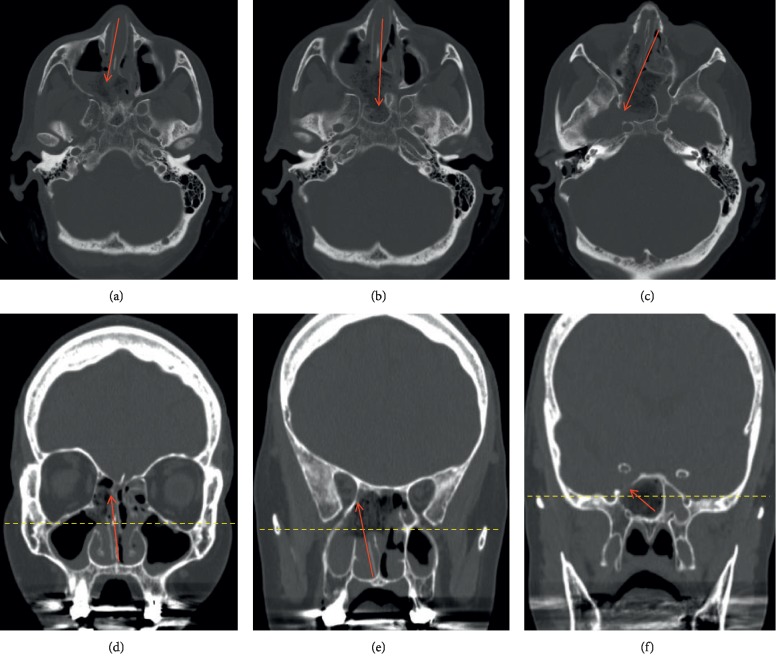
CT maxillofacial with sequential cuts to demonstrate the overall trajectory of an endoscopic endonasal right transpterygoid approach. (a–c) Axial views of the anterior skull base. (d–f) Coronal views of the same region. Red arrows estimate the working trajectory. Note the bony opening in the anteromedial aspect of the middle cranial fossa to expose the contents of MC in axial (c) and coronal views (f). This corresponds to the intraoperative view in Figure 4 with the internal carotid artery located superior and medial to the bony opening.

An endoscopic endonasal approach, importantly, provides an anteromedial corridor to the trigeminal ganglion with reduced need for trigeminal nerve manipulation required to maximize operative views (Figure 9). Several expanded variations of the endoscopic endonasal approach to MC have been described with varying success, including the transpterygoid and transmaxillary approaches [12]. Particularly for trigeminal schwannomas in MC, the endoscopic endonasal transpterygoid approach has shown promising ability to achieve complete resection with minimal morbidity [25]. Alternatively, a transmaxillary route provides a safe working angle in conjunction with the transphenoidal route [23]. The feasibility of this transmaxillary approach to MC has moreover been substantiated in cadaveric specimens. Notably, as the endonasal approach is laterally extended, the contents of the infratemporal fossa begin to encroach on the operative corridor, making visualization and instrumentation more difficult [12, 26].

As epidermoid cysts are exceedingly rare intracranial lesions, documentation of successful operation on them in Meckel's cave via an endoscopic endonasal approach is limited. Endoscopic-assisted techniques have been shown to be useful in the visualization of epidermoid tumors in several studies [27, 28]. In addition, the endoscopic endonasal corridor has been utilized to resect epidermoid cysts from other locations along the cranial base [29–32]. However, to our knowledge, there have only been two reports of this approach for the successful resection of an epidermoid cyst in MC by a purely endoscopic endonasal approach, each describing just one adult patient [12, 33]. According to Best et al., this minimally invasive approach for removal of a cyst in the middle cranial fossa (that involved Meckel's cave) may also have potentially easier surveillance of future epidermoid recurrence [33].

In both of our cases, near-complete resection was achieved based on intraoperative impression and neuronavigational confirmation. Minor residual epidermoid remained in both cases, but postoperative MRI completed within 48 hours of surgery revealed no appreciable residual. Both patients were discharged from the hospital on postoperative day three without any major complications. Minor, transient neurologic deficits/symptoms relating to CN V (V2 pain) and VI (mild abducens palsy) were noted in patients 1 and 2, respectively. Transient V or VI deficits have commonly been described following resection of MC tumors through craniotomy approaches [8], likely due to trigeminal and abducens nerve manipulation, respectively, during the resection. These symptoms were resolved at six-week follow-up in both patients, and one-year follow-up reveals continued clinical and radiographic stability. Our plan for the immediate future is to continue to follow these patients with annual MR imaging and clinical examination to evaluate for recurrence.

While only limited to two cases, our results suggest that the endoscopic endonasal approach to MC utilizing a transpterygoid approach may represent a safe alternative to open surgical approaches for epidermoid cysts at this region. Particularly, in our second case, an open craniotomy approach would have likely been associated with a lower rate of resection, given the marked anterior extension of the epidermoid and narrow working corridor. Given the proximity of the cranial nerve III through VI and the carotid artery, there is still potential for neurovascular injury with the endonasal approach. Utilization of image guidance to confirm intraoperative findings, the usage of neurophysiological monitoring to provide real-time feedback, judicious use of intraoperative ultrasound, and careful review of the preoperative imaging can help minimize such injuries. Additionally, such cases should only be considered and performed by multidisciplinary teams of neurosurgeons and otolaryngologists that are very experienced with endoscopic endonasal skull base surgery.

## 4. Conclusion

The endoscopic endonasal approach can be utilized for the safe and effective resection of MC epidermoid cysts. Intraoperative neurophysiological monitoring is very helpful in these particular cases. These approaches should only be considered by experienced, multidisciplinary teams following careful review of preoperative imaging. Additional follow-up and studies will be needed to determine long-term outcomes.
